# Improving the built environment in urban areas to control *Aedes aegypti*-borne diseases

**DOI:** 10.2471/BLT.16.189688

**Published:** 2017-06-09

**Authors:** Steve W Lindsay, Anne Wilson, Nick Golding, Thomas W Scott, Willem Takken

**Affiliations:** a Department of Biosciences, Durham University, South Road, Durham, DH1 3LE, England.; b School of BioSciences, University of Melbourne, Melbourne, Australia.; c Department of Entomology and Nematology, University of California Davis, Davis, United States of America.; d Laboratory of Entomology, Wageningen University, Wageningen, Netherlands.

The vector *Aedes aegypti* is now present in nearly every tropical and sub-tropical region in the world and poses a threat to health globally. The mosquito can transmit several viruses that cause diseases, such as dengue fever, chikungunya, yellow fever and Zika virus infection. Recent outbreaks of *Ae. aegypti*-borne diseases have shown that urban areas are particularly vulnerable because the built environment provides ideal conditions for mosquito proliferation and contact with humans. Unless the global public health community takes a coordinated, pre-emptive approach to controlling the *Ae. aegypti* population, these outbreaks will become more common and widespread as urban populations expand and movement of people and their goods increase. Improving the built environment would contribute to a long-term solution to reducing the threat of *Ae. aegypti*-borne diseases.

Our ability to deal with *Ae. aegypti*-borne viral epidemics is limited. Apart from supportive care, specific treatments for vector-borne viral diseases are lacking. No commercial vaccines for Zika or chikungunya are available, the only licensed dengue vaccine is partially protective^1^ and globally the yellow fever vaccine is in short supply.^2^ Although current vector control programmes are often poorly resourced and under-used,^3^ historically, vector control was the main method for controlling mosquito-borne diseases. By using container inspections, oiling of breeding sites and later perifocal spraying of DDT (dichlorodiphenyltrichloroethane) in water containers and on nearby walls, *Ae. aegypti*, yellow fever and dengue fever were successfully eliminated from much of South America in the 1960s.^4^ In the 1970s and 1980s in Singapore and in the 1980s and 1990s in Cuba, controlling adult and larval *Ae. aegypti* reduced dengue transmission. In the future, new methods of vector control, such as novel delivery systems for insecticides with new modes of action and release of *Wolbachia-*infected or genetically-modified mosquitoes, may contribute to the control or elimination of mosquito-borne diseases.^5^ Affected towns and cities, however, already have several options to reduce *Ae. aegypti*-borne diseases and these options should be built into future planning strategies.

Current *Ae. aegypti* control focuses on reducing densities of immature and adult mosquitoes with larvicides or adult insecticides. While these interventions can be effective, continued reliance on these single-intervention control programmes is resource-intensive and threatened by insecticide resistance. The World Health Organization^3^^,^^6^ and other major international organizations^7^ have recommended an intersectoral approach to achieve more effective and sustainable vector control. Governments, however, have often overlooked such approaches when designing vector control programmes.

An underutilized aspect of integrated vector management is improving the urban built environment to reduce *Ae. aegypti* populations and their contact with humans.^8^ The built environment in many urban areas provides abundant habitats for the immature stages of *Ae. aegypti*, and high human population densities create the potential for large outbreaks of *Aedes*-borne diseases. More than half of the world’s population currently lives in urban areas and by 2050 it is estimated that 70% of the population will live in cities.^9^ This urban expansion will increase the frequency and intensity of *Aedes*-borne outbreaks. However, developing urban areas that minimize human contact with mosquitoes could enable sustainable and cost–effective prevention of mosquito-borne diseases.

Several aspects of urban planning can be targeted to reduce human contact with *Ae. aegypti*. Reducing the availability of small plastic containers around homes and improving solid waste management will remove habitats for *Ae. aegypti* larvae development. Provision of constant piped water will reduce the need to store water in containers in and around homes, since water-filled containers are known to be favoured habitats for *Ae. aegypti*. Houses can be designed to prevent adult mosquitoes from entering, either by sealed or screened openings. Urban planning to reduce vector proliferation and human contact can only be successful if it is combined with community engagement, so that communities understand the diseases transmitted by these vectors and contribute to control efforts.

Reducing vector densities would not only reduce the chance of future epidemics occurring, but also reduce the biting nuisance by other urban pest mosquitoes. Furthermore, most improvements that reduce human contact with mosquitoes will have a range of other social and health benefits by improving the domestic environment, such as easy access to potable water and more sanitary living conditions. Delivering these urban improvements should not only be seen as gains for urban development, but also as a supplementary vector-control intervention to those interventions delivered through the health sector, such as space spraying or larviciding.

We argue that urban improvements that reduce the mosquito population should be a component of future *Ae. aegypti*-borne disease prevention strategies, and be seen as an important component of sustainable development. This approach is closely aligned with the sustainable development goals (SDGs), particularly SDG 11 and 17. SDG 11 demands action to “make cities and human settlements inclusive, safe, resilient and sustainable” through improvements to housing and basic services. SDG 17 calls for “sustainable development through global partnerships” and building out *Ae. aegypti* will require close collaboration between governments, the private sector and civil society.

A policy framework exists through which these changes can be implemented. The *New urban agenda*, which is a product of the SDGs, was adopted by the United Nations (UN) conference on housing and sustainable development (Habitat III) in Quito, Ecuador in 2016 and subsequently endorsed by the UN General Assembly.^10^ The agenda is an attempt to readdress the way in which towns and cities are planned, designed, financed, developed, governed and managed to make them more resilient and sustainable. Specifically, the agenda recognizes that urban centres, particularly in developing countries, are vulnerable to environmental risks including those from vector-borne diseases and therefore promotes disaster risk reduction and management.^10^

To build settlements that are resilient against *Ae. aegypti*-borne diseases, vector control experts should reach out and work with those who plan and design the built environment. By creating a safe, reliable and protective water supply system, removing domestic waste and sealing or screening homes, we can hinder immature mosquito development and reduce biting densities of mosquitoes. Creating environments unfavourable for *Ae. aegypti* must be a priority when building safe, resilient and sustainable towns and cities.
